# *Imperata cylindrica* (L.) Raeusch.: a narrative review of phytochemistry, pharmacology, ethnopharmacology, botany, and comprehensive utilization

**DOI:** 10.3389/fphar.2026.1875167

**Published:** 2026-07-14

**Authors:** Jianing Li, Chengchen Wang, Hengzheng Zhan, Xiaoyuan Wang, Wei Guan, Yu Zhao, Zhenpeng Xu

**Affiliations:** 1 State Key Laboratory of Discovery and Utilization of Functional Components in Traditional Chinese Medicine, Key Lab of Chemical Biology (MOE), School of Pharmaceutical Sciences, Cheeloo College of Medicine, Shandong University, Jinan, China; 2 Key Laboratory of Basic and Application Research of Beiyao (Heilongjiang University of Chinese Medicine), Ministry of Education, Harbin, China

**Keywords:** anti-inflammation, diuresis, ethnopharmacology, Imperata cylindrica, phytochemistry

## Abstract

Imperata cylindrica is a perennial botanical drug with both ecological impacts and diverse utilization values. As a traditional Chinese medicine, its rhizome is widely applied in traditional medical systems of various countries, which can cool blood to arrest bleeding and clear away heat to induce diuresis. Modern research has isolated and characterized 141 chemical metabolites from *Imperata cylindrica*, mainly including flavonoids and triterpenoids. These metabolites have been associated with multiple bioactivities, including anti-inflammatory, antioxidant, and immunomodulatory effects, and many proprietary Chinese medicines containing its rhizome have been applied in clinical practice. *Imperata cylindrica* also shows good application prospects in food processing, agriculture and animal husbandry, and ecological restoration. However, current research is limited by insufficient characterization of active metabolites, inadequate studies on isolated metabolites, and a predominance of *in vitro* and preclinical evidence. In the future, efforts should focus on the in-depth development and pharmacological verification of active metabolites, improving the cell-animal experimental system, promoting industrial application in multiple fields, and counteracting its invasion status through resource utilization to achieve a win-win situation of ecology and economy.

## 1 Introduction

*Imperata cylindrica* (L.) Raeusch. is a common perennial botanical drug, belonging to the Poaceae family. *Imperata cylindrica* was recorded in a literary work called “The *Book of Songs*” from the 6th century BC. It not only possesses the poetic imagery described in the book, such as “White flowered rushes sway, together with white grass (*Imperata cylindrica*)” (Xu, 2011), “from the pasture lands she gives me a shoot of the white grass, truly elegant and rare” (Legge, 2014), but also might become an “ecological tyrant” and a “farmland nightmare” due to its strong vitality and reproductive ability. For this abundant natural resource, how to utilize it rationally is the key to harmoniously coexisting with nature. From the line “An antelope is killed, and wrapped in white afield” in the Book of Songs, we can know that people had begun to utilize it as early as two thousand years ago. Apart from using it to wrap food, people later also used it to build thatched houses, as well as to make pot cover, baskets and other utensils.

What is more worthy of attention is that as people began to use the *I. cylindrica* (*Imperata cylindrica*), they gradually discovered its medicinal value. Especially its rhizome, it is now one of the most commonly used traditional Chinese medicines for treating inflammatory diseases of the digestive system and urinary system. The rhizome of *I. cylindrica* (Imperatae Rhizoma) used for medicinal purposes, known as “Baimaogen”, was first recorded in *Shennong’s Classic of Materia Medica.* It was extensively used in traditional medicine to cool the blood for hemostasis and to clear heat to promote diuresis documented in *Pharmacopoeia of the People’s Republic of China* (2025 edition). It is indicated for the treatment of hematemesis and hematuria due to blood-heat, polydipsia in febrile diseases, jaundice resulting from dampness-heat, edema with oliguria, and heat strangury with painful urination. Furthermore, in traditional medicine systems of India, Japan, Korea, and other regions, *I. cylindrica* is also used as a medicinal botanical drug and demonstrates good efficacy when combined with other botanical drugs to treat dysentery, jaundice, malaria and kinds of inflammations. Due to the benefits of *I. cylindrica* in respiratory and urinary system diseases, along with its high safety profile, it is also used as a health food. At the same time, it is also an excellent livestock feed.

In recent years, research on *I. cylindrica* has continuously increased, with numerous studies confirming its important pharmacological effects, including anti-inflammatory, antioxidant, immunomodulatory, antitumor, hemostatic, and diuretic activities, as well as hepatoprotective and nephroprotective properties, which demonstrate its broad development value. These diverse pharmacological actions originate from its natural active metabolites, which represent a breakthrough point for the development of new and highly effective drugs. Researchers have currently isolated and identified 141 chemical metabolites, covering various types including chromones, triterpenoids, phenylpropanoids, aromatic metabolites, and other metabolites. For example, cylindrin alleviates renal fibrosis by inhibiting M2 macrophage polarization, while arundoin significantly suppresses the proliferation of prostate cancer cells and induces their apoptosis (Chen, 2016; 2017; Li et al., 2023). Additionally, based on the hemostatic effects of *I. cylindrica*, drugs such as Peitu Qingxin Granules, Qingre Tonglin Granules have been developed and marketed.

In rural areas, *I. cylindrica* is still used for weaving household utensils and repairing thatched roofs. It has also been incorporated into beverages and food products. However, its ecological destructiveness as an invasive species cannot be ignored. Therefore, a comprehensive review is needed to clarify its medicinal, ecological, and industrial value and to support its rational utilization.

## 2 Methodology

A comprehensive literature search was conducted using Web of Science, PubMed, Elsevier, SpringerLink, Wiley Online Library, CNKI, Wanfang, VIP, and Baidu Scholar for publications from 1960 to 2025. Search terms included “*Imperata cylindrica*,” “Imperatae Rhizoma,” “Baimaogen,” “cogongrass,” “phytochemistry,” “pharmacology,” “ethnopharmacology,” “toxicity,” and “utilization.” A total of over 400 publications from 1960 to November 2025 were initially identified, ultimately, 104 publications were cited in this review.

Inclusion criteria: (1) peer-reviewed original research articles, reviews, and academic dissertations; (2) studies reporting clear botanical sources, chemical metabolites, pharmacological effects, quality control, or clinical applications of *I. cylindrica*; (3) papers with detailed experimental methods and reliable results. Exclusion criteria: duplicate publications, abstracts without full text, conference summaries without data support, non-peer-reviewed grey literature, and studies with unclear experimental materials or unauthenticated plant sources. The literature was screened by title, abstract, and full text to ensure accuracy, methodological rigor, and relevance. Extracted information was categorized according to botany, traditional use, chemical metabolites, pharmacological evidence, safety, quality control, and comprehensive utilization, followed by assessment of evidence strength and methodological limitations.

## 3 Botany

**FIGURE 1:**
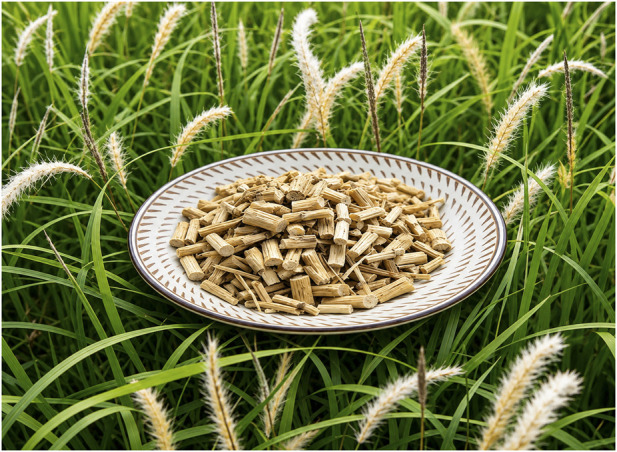
*Imperata cylindrica* and its main medicinal part (Imperatae Rhizoma).

*Imperata cylindrica* is a perennial herbaceous plant of the Poaceae family, possessing stout, elongated rhizomes (Figure 1). The culms are erect, 30–80 cm tall, with 1–3 nodes; the nodes are glabrous. The leaf sheaths are clustered at the culm base, considerably longer than their internodes, thick in texture, and becoming fibrous and fragmenting with age; the ligule is membranous, about 2 mm long, appressed to the back or with soft hairs at the sheath mouth; the basal (tiller) blades are about 20 cm long, about 8 mm wide, flat, and relatively thin in texture; the culm leaves are 1–3 cm long, narrowly linear, often involute, tapering to a sharp tip, narrowing downwards, sometimes with a petiole, hard in texture, glaucous, and softly hairy on the upper surface near the base. The panicle is dense, 20 cm long, up to 3 cm wide; the spikelets are 4.5–5 (-6) mm long, with a basal disc bearing silky hairs 12–16 mm long; the two glumes are herbaceous with hyaline margins, nearly equal, with 5–9 veins, tapering or slightly obtuse at the apex, often ciliate, with sparsely long silky hairs between the veins; the first lemma is ovate-lanceolate, about two-thirds the length of the glumes, hyaline-membranous, veinless, pointed or toothed at the apex; the second lemma is nearly equal to its palea, about half the length of the glumes, ovate, toothed and ciliate at the apex; there are 2 stamens, with anthers 3–4 mm long; the styles are slender, more or less united at the base, with 2 stigmas, purplish-black, plumose, about 4 mm long, exserted from the apex of the spikelet. The caryopsis is elliptical, about 1 mm long, with the embryo about half the length of the caryopsis. Flowering and fruiting period: April to June.

*Imperata cylindrica* is primarily distributed in regions including North Africa, Turkey, Iraq, Iran, Central Asia, the Caucasus, the Mediterranean area, and Northern China (Editorial Committee of Flora of China, 1997). It thrives in sunlight but tolerates partial shade, prefers fertile conditions yet is highly tolerant of poor soil, and favors loose, moist soil. It is notably tolerant of waterlogging as well as drought, adapting to various soil types such as clay, sand, and loam. It grows most abundantly in loose, sandy soils, where its propagation is most vigorous and its impact as a weed is most severe. It inhabits grasslands along rivers in low-altitude mountainous areas and plains, farmlands, orchards, nurseries, field edges, roadsides, wastelands, grassy slopes, forest margins, open woodlands, shrublands, ditches, riverbanks, embankments, lawns, sandy meadows, deserts, and coastal areas, demonstrating extremely strong competitive and spreading ability (He and Geng, 2023; Xu, 2008).

## 4 Ethnopharmacology

*Imperata cylindrica* has a long history of application in human health. Originating in Asia, *I. cylindrica* is now widely distributed across many parts of the world. In China, the rhizome of *Imperata cylindrica* (L.) Raeusch. (Imperatae Rhizoma) is known as “Baimaogen” and is used as an important traditional Chinese medicine for its diuretic, heat-clearing, blood-cooling, and hemostatic properties in India, it is known as “Dharbha” and serves as a key component in traditional remedies, used to treat ailments such as dysentery, jaundice, and skin infections (Subramaniam and Sivasubramanian, 2015). In Vietnamese traditional medicine, it is commonly used as a diuretic. In Malagasy traditional medicine, a decoction made from the its aerial parts is employed to relieve pain and treat malaria, while a decoction of the rhizome is used for colic, tonsillitis, and laryngitis (Razafindrakoto et al., 2025).

It was first recorded in the *Shennong’s Classic of Materia Medica* (神农本草经, the Qin and Han Dynasties) in China, where it was classified as a middle-grade botanical drug. Imperatae Rhizoma is cold in nature and sweet in flavor, with a channel tropism to the Lung, Stomach, and Bladder meridians. Botanical drugs with a cold nature primarily clear heat, purge fire, cool the blood, and resolve toxicity, making them suitable for treating heat-pattern disorders. Its channel tropism indicates that Imperatae Rhizoma mainly acts on the upper jiao (Lung), middle jiao (Stomach), and lower jiao (Bladder). It can clear heat from the Lung and Stomach and promote diuresis to relieve strangury, thereby achieving a synergistic effect of “clearing the upper and disinhibiting the lower”. Therefore, it can treat inflammatory diseases of the respiratory, digestive, and urinary systems. For cooling blood and stopping bleeding, the carbonized form of it (Maogen Tan) is more effective *The Thousand Gold Formulas* (千金方,the Tang Dynasty) states that its juice can be taken to relieve alcohol toxicity. *The Handbook of Prescriptions for Emergencies* (肘后备急方,the Jin Dynasty) mentions its use in treating five types of jaundice and dysuria due to edema. *The Supplement to Recipes Worth A Thousand Gold* (千金翼方,the Tang Dynasty) records that a handful of Imperatae Rhizoma can be used to treat persistent hematemesis. Thus, its core therapeutic actions are to cool the blood for hemostasis and to clear heat to promote diuresis, making it applicable for treating heat-pattern conditions such as cough due to lung heat, retching and hiccup resulting from stomach heat, and urinary tract infections.

Imperatae Rhizoma serves as a principal ingredient in several traditional formulas, including Baimaogen Tang and Liangxue Wugen Tang, where it exerts the therapeutic effect to clear heat and cool the blood. It can also function as a minister ingredient in formulas such as Baimao San, assisting the principal ingredient and producing synergistic effects (see Table 1 for specific formulas). Other representative formulas include “Er Xian Yin”, composed of Imperatae Rhizoma and Nelumbinis Rhizomatis Nodus (the rhizome of *Nelumbo nucifera* Gaertn. [Nelumbonaceae]), is combined with Rubiae Radix et Rhizoma (the root and rhizome of *Rubia cordifolia* L. [Rubiaceae]) to treat patients with blood heat and hematuria, cooling blood and stopping bleeding without causing stasis (Xue et al., 2016).“Qing Re Chu Shi Tang”, developed by the renowned TCM (traditional Chinese medicine) dermatologist Professor Zhao Bingnan, is a clinically common empirical formula for treating skin diseases caused by damp-heat. It consists of Imperatae Rhizoma, Gentianae Radix et Rhizoma (the rhizome and root of *Gentiana manshurica* Kitag. [Gentianaceae], *Gentiana scabra* Bunge [Gentianaceae] and *Gentiana triflora* Pall. [Gentianaceae]), Scutellariae Radix (the root of *Scutellaria baicalensis* Georgi [Lamiaceae]), Rehmannia Radix (the root of *Rehmannia glutinosa* (Gaertn.) Libosch. ex DC. [Orobanchaceae]), Gypsum Fibrosum (mainly contains CaSO_4_•2H_2_O), Isatidis Folium (the leaves of *Isatis tinctoria* L. [Brassicaceae]), Plantaginis Herba (the whole plant of *Plantago asiatica* L. [Plantaginaceae] and *Plantago depressa* Willd. [Plantaginaceae]) and Liuyi Sɑn (composed of Talcum and the root and rhizome of *Glycyrrhiza glabra* L. [Fabaceae], *Glycyrrhiza uralensis* Fisch. ex DC. [Fabaceae] and *Glycyrrhiza inflata* Batalin [Fabaceae]), has the effects of clearing heat, eliminating dampness, cooling blood, and detoxifying. It is clinically used to treat acute eczema, allergic dermatitis, herpes zoster, and other skin conditions with good efficacy (Yang et al., 2019). Studies have shown that postoperative anal fistula patients treated with Fufuxin Liquid combined with Ginseng and Imperatae Rhizoma Decoction,which composed of Imperatae Rhizoma, Scutellariae Radix (the root of *Scutellaria baicalensis* Georgi [Lamiaceae]), Angelicae Sinensis Radix (the root of *Angelica sinensis* (Oliv.) Diels [Apiaceae]), Pheretima [*Pheretima aspergillum* (E.Perrier, 1872), *Pheretima vulgaris* (chen 1930)*, Pheretima guillelmi* (Michaelsen, 1895) *and Pheretima pectinifera* (Michaelsen, 1931)], Ginseng Radix et Rhizoma (the root and rhizome of *Panax ginseng* C.A.Mey. [Araliaceae]) and Honey-processed the root and rhizome of *Glycyrrhiza glabra* L. [Fabaceae], *Glycyrrhiza uralensis* Fisch. ex DC. [Fabaceae] and *Glycyrrhiza inflata* Batalin [Fabaceae]), exhibited favorable outcomes, with reduced serum levels of tumor necrosis factor-α (TNF-α), interleukin-1β (IL-1β), and interleukin-6 (IL-6) (Li et al., 2024). Other applications, such as Imperatae Rhizoma decoction combined with Lamivudine for chronic hepatitis B (Zhan, Shen, and Li, 2018), and ascites fire therapy combined with Imperatae Rhizoma Soup for ascites due to liver disease (Wang, 2015), have also demonstrated promising therapeutic effects. The flowers of *I. cylindrica* are also used in traditional medicine. Its flowers are used to arrest bleeding. The Quanzhou Materia Medica records that it can treat epistaxis. Modern experiments have demonstrated that its flowers can shorten coagulation and bleeding time in rabbits (Department of Biochemistry, 1960). Upon analysis, the “*Imperata cylindrica* spike”, which is the unopened inflorescence-also exhibits good hemostatic activity.

**TABLE 1:** The prescriptions of *Imperata cylindrica*.

Prescriptions	Main compositions (except for Imperata cylindrica)	Traditional uses	References
Baihe Yinzi	*Morus alba* L. [Moraceae] (root bark) *Tetrapanax papyrifer* (Hook.) K. Koch [Araliaceae] (pith) *Lilium lancifolium* Thunb. [Liliaceae], *Lilium brownii* var. viridulum Baker [Liliaceae] and *Lilium pumilum* Redouté. [Liliaceae] (fleshy scale leaf)	Draining Lung Fire, strangury	Jin Gui Yi (金匮翼)
Baibiandou San	Lablab purpureus (L.) Sweet [Fabaceae] (seed) *Zingiber officinale* Roscoe. [Zingiberaceae](rhizome) *Eriobotrya japonica* (Thunb.) Lindl. [Rosaceae] (leaf) *Panax ginseng* C. A. Mey. [Araliaceae] (root and rhizome) Pinellia ternata (Thunb.) Makino [Araceae] (processed products) *Atractylodes macrocephala* Koidz. [Asteraceae] (rhizome)	Lung Wilting and Anorexia with Thoracic Fullness due to Chronic Hemoptysis and Profuse Saliva	Syndrome of Pearl Pills (普济本事方)
Baimaogen San	*Silybum marianum* (L.) Gaertn. [Asteraceae] (root) *Scutellaria baicalensis* Georgi [Lamiaceae] (root) *Morus alba* L. [Moraceae] (root bark) *Aster tataricus* L.f. [Asteraceae] (root and rhizome) *Rhinoceros unicornis* L., 1758, *Rhinoceros sondaicus* Desmarest, 1822, *Dicerorhinus sumatrensis* (G.Fischer, 1814), *etc.*, (horn)	Bloody sputum caused by congested heat in the Heart and Lungs	Taiping Shenghui Prescription (太平圣惠方)
Baimaogen Tang	*Musa basjoo* Siebold ex Miq. [Musaceae] (rhizome) *Talcum* *Alternanthera sessilis* (L.) DC. [Amaranthaceae] (whole plant)	Heat Strangury with painful urination and unremitting fever	Taiping Shenghui Prescription (太平圣惠方)
Kuigen Yinzi	*Malva verticillata* L. [Malvaceae] (root) *Talcum* *Ampelopsis glandulosa* var. heperophylla (Thunb.) Momiy. [Vitaceae] (root bark) Dianthus chinensis L. [Caryophyllaceae] and Dianthus superbus L. [Caryophyllaceae] (aerial part)	Retention of urine in women	Taiping Shenghui Prescription (太平圣惠方)
Rusheng San	Ophiopogon japonicus (Thunb.) Ker Gawl. [Asparagaceae] (root) Descurainia sophia (L.) Webb ex Prantl [Brassicaceae] and *Lepidium apetalum* [Brassicaceae] Willd. (seed) *Plantago asiatica* L. [Plantaginaceae] and *Plantago depressa* Willd. [Plantaginaceae] (seed) *Santalum album* L. [Santalaceae] (heartwood) Iris oxypetala Bunge [Iridaceae] (flower) *Forsythia suspensa* (Thunb.) Vahl [Oleaceae] (fruit)	Urinary stone	Pu Ji Fang (普济方)
Fupen Yin	*Rubus chingii* Hu [Rosaceae] (fruit) *Panax ginseng* C. A. Mey. [Araliaceae] (root and rhizome) *Juncus effusus* L. [Juncaceae] (pith) Pinellia ternata (Thunb.) Makino [Araceae] (processed products) *Peucedanum praeruptorum* (Dunn) Pimenov [Apiaceae] (root) *Atractylodes lancea* (Thunb.) DC. [Asteraceae] (rhizome)	Disharmony of Stomach Qi presenting with retching and the refusal of food	Sheng Ji Zong Lu (圣济总录)
Baimao San	*Syzygium aromaticum* (L.) Merr. and L.M.Perry [Myrtaceae] (flower bud) *Morus alba* L. [Moraceae] (leaf) *Panax ginseng* C. A. Mey. [Araliaceae] (root and rhizome) Pogostemon cablin (Blanco) Benth. [Lamiaceae] (leaf)	Unremitting vomiting and regurgitation	Xiaoer Weisheng Zongwei Lunfang (小儿卫生总微论方)
Liangxuewugen Tang	Rubia cordifolia L. [Rubiaceae] (root and rhizome) Arnebia euchroma (Royle ex Benth.) I.M.Johnst. [Boraginaceae] (root) *Isatis tinctoria* subsp. Tinctoria [Brassicaceae] (root) Trichosanthes kirilowii Maxim. [Cucurbitaceae] and *Trichosanthes rosthornii* Harms [Cucurbitaceae] (fruit)	Invigorating the blood and resolving stasis	Zhao Bingnan’s Clinical Experience Collection (赵炳南临床经验集)
Jianggong Tang	*Polistes olivaceus* (Deg., 1773), *Polistes jokahamae* Radoszkowski, 1887, and *Parapolybia varia* (Fabricius, 1787) (nest) Charred human hair	Abdomino-hypochondriac pain and hypogastric distention with heat strangury resulting from bladder heat	Ruizhutang Experience Prescription (瑞竹堂经验方)

Imperatae Rhizoma is also utilized in modern Chinese patent medicines as a key component. Several preparations, such as Peitu Qingxin Granules, Lixuekang Soft Capsules, and Qingre Tonglin Granules, incorporate it to exert its effects of clearing heat, eliminating dampness, stopping bleeding, and cooling the blood.

## 5 Phytochemistry

To date, 141 distinct metabolites have been isolated and identified from *Imperata cylindrica*, encompassing chromones, triterpenoids, phenylpropanoids, simple aromatic metabolites, and other metabolites.

It is noteworthy that the vast majority of current research focuses on the rhizomes of *I. cylindrica*, with relatively few studies dedicated to the isolation and identification of metabolites from its aerial parts. Among the metabolites cited in this paper, thirteen originate from the aerial parts. Studies have shown that three of these 13 metabolites: 2-methoxyestrone, 11,16-dihydroxypregn-4-ene-3,20-dione, and tricin, exhibit growth-inhibitory effects on breast cancer and colon cancer cells. Stigmast-4-en-3-one demonstrates analgesic and anti-inflammatory activities, while isovanillin exhibits antioxidant activity. Jaceidin, quercetagetin-3,5,6,3′-tetramethyl ether and *β*-sitosterol-3-O-*β*-D-glucopyranosyl-6′-tetradecanoate show strong hepato-protective activity. Furthermore, the application of GC-MS analysis has led to the characterization of 12 metabolites with various pharmacological activities from the leaves (Hazarika and Ahmed, 2025). This undoubtedly indicates that the aerial parts hold broad research prospects, and their pharmacological effects await more in-depth exploration.

### 5.1 Chromones

**FIGURE 2:**
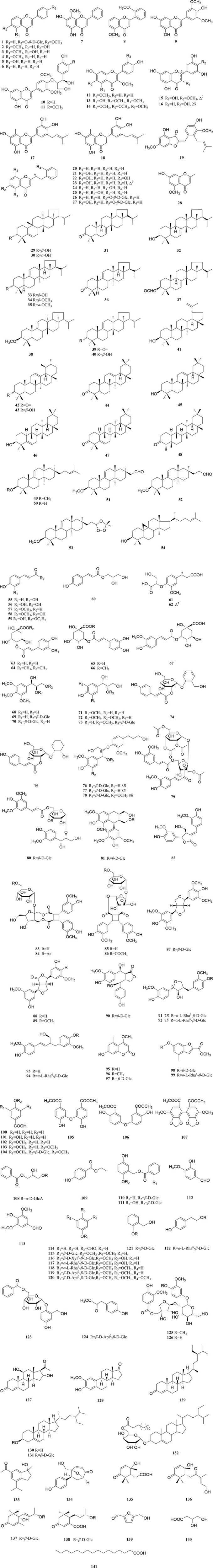
Structures of metabolites isolated from *Imperata cylindrica*.

To date, 19 flavonoid metabolites (1–19 in Figure 2; Table 2) have been isolated from *I. cylindrica*, including flavones, flavonols, dihydroflavones, and aurones. Furthermore, it has found that prenylated flavonoids (17–19) which have unique carbon skeleton (Nago et al., 2021). Besides, eight phenylethylchromone (20–27) have also been isolated from *I. cylindrica*.

**TABLE 2:** The metabolites isolated from *Imperata cylindrica*.

NO.	Metabolites	Extracts	Parts	Molecular formula	CAS Number	References
1	4′-methoxy-flavone-6-O-*β*-D-glucopyranoside	70%EtOH	Rhizome	C_22_H_22_O_9_	1,588,495–01-5	Liu et al. (2013b)
2	4′-hydroxy-5-methoxyflavone	70%EtOH	Rhizome	C_16_H_12_O_4_	106,848–87-7	Liu et al. (2013b)
3	6-hydroxy-5-methoxyflavone	70%EtOH	Rhizome	C_16_H_12_O_4_	118,021–60-6	Liu et al. (2013a)
4	5-methoxyflavone	70%EtOH	Rhizome	C_16_H_12_O_4_	42,079–78-7	Liu et al. (2013a)
5	5-hydroxyflavone	70%EtOH	Rhizome	C_16_H_12_O_3_	491–78-1	Liu et al. (2013b)
6	flavone	70%EtOH	Rhizome	C_15_H_10_O_2_	525–82-6	Liu et al. (2013b)
7	5,7- dihydroxy-8-methoxyflavone	70%EtOH	Rhizome	C_16_H_12_O_5_	632–85-9	Liu et al. (2013a)
8	5,2′-dimethoxyflavone	70%EtOH	Rhizome	C_17_H_14_O_4_	6,697–62-7	Jiang (2021)
9	tricin	70%MeOH	Aerial parts	C_17_H_14_O_7_	520–32-1	Mohamed et al. (2009)
10	tricin 4′-O-(erythro-*β*-4-hydroxyphenylglyceryl) ether	70%EtOH	Rhizome	C_26_H_24_O_10_	65,870–44-2	Jiang (2021)
11	tricin 4′-O-(erythro-*β*-guaiacylglyceryl) ether	70%EtOH	Rhizome	C_27_H_26_O_11_	369,390–52-3	Jiang (2021)
12	3,5-di-O-methyl-kaempferol	70%MeOH	Aerial parts	C_17_H_14_O_7_	1,486–66-4	Mohamed et al. (2009)
13	jaceidin	70%MeOH	Aerial parts	C_18_H_16_O_8_	10,173–01-0	Mohamed et al. (2009)
14	quercetagetin-3,5,6,3′-tetramethyl ether	70%MeOH	Aerial parts	C_19_H_18_O_8_	58,130–92-0	Mohamed et al. (2009)
15	mearnsetin	MeOH	Plant	C_16_H_12_O_8_	16,805–10-0	Nago et al. (2021)
16	3′,4′,5,5′,7-pentahydroxyflavanone	MeOH	Plant	C_15_H_12_O_7_	857,824–01-2	Nago et al. (2021)
17	cylindricine B	MeOH	Plant	C_21_H_20_O_6_	​	Nago et al. (2021)
18	cylindricine A	MeOH	Plant	C_21_H_22_O_6_	​	Nago et al. (2021)
19	cylindraucine	MeOH	Plant	C_22_H_22_O_6_	​	Nago et al. (2021)
20	flidersiachromone	MeOH	Rhizome	C_17_H_14_O_2_	61,828–53-3	Yoon et al. (2006)
21	5-hydroxy-2-(2-phenylethyl)chromone	MeOH	Rhizome	C_17_H_14_O_3_	877,673–99-9	Yoon et al. (2006)
22	5-hydroxy-2-[2-(2-hydroxyphenyl)ethyl]chromone	MeOH	Rhizome	C_17_H_14_O_4_	357,637–15-1	Yoon et al. (2006)
23	5-hydroxy-2-styrylchromone	MeOH	Rhizome	C_17_H_14_O_2_	158,264–61-0	Yoon et al. (2006)
24	8-hydroxy-2-(2-phenylethyl) chromone	70%EtOH	Rhizome	C_17_H_14_O_3_	1,588,494–99-8	Liu et al. (2013b)
25	6-hydroxy-2-(2-phenylethyl) chromone	70%EtOH	Rhizome	C_17_H_14_O_3_	84,294–90-6	Jiang (2021), Jiang et al. (2022)
26	2-(2-phenylethyl)chromone-8-O-*β*-D-glucopyranoside	70%EtOH	Rhizome	C_23_H_24_O_8_	1,588,495–00-4	Liu et al. (2013b)
27	5-hydroxy-8-O-*β*-D-glucopyranosyl-2-(2-phenylethyl) chromone	70%EtOH	Rhizome	C_23_H_24_O_9_	​	Jiang (2021), Jiang et al. (2022)
28	isoeugenin	MeOH	Rhizome	C_11_H_10_O_4_	3,449–40-9	An et al. (2015)
29	simiarenol	Hexane/Benzene	Rhizome	C_30_H_50_O	1,615–94-7	(Nishimoto et al., 1968)
30	3-episimiarenol	n-hexane	Rhizome	C_30_H_50_O	2,605–19-8	Sakai et al. (2023)
31	14-epiarbor-7-en-3-one	n-hexane	Rhizome	C_30_H_48_O	​	Sakai et al. (2018)
32	14-epiarbor-7-en-3*β*-ol	n-hexane	Rhizome	C_30_H_50_O	​	Sakai et al. (2018)
33	isoarborinol	Hexane/Benzene	Rhizome	C_30_H_50_O	5,532–41-2	(Nishimoto et al., 1968)
34	cylindrin	Hexane/Benzene	Rhizome	C_31_H_52_O	17,904–55-1	(Nishimoto et al., 1968)
35	arborinol methyl ether	n-hexane	Rhizome	C_31_H_52_O	25,279–40-7	Sakai et al. (2023)
36	arborinone	n-hexane	Rhizome	C_30_H_48_O	25,465–81-0	Sakai et al. (2023)
37	14-epiarbor-7-en-3*β*-yl	n-hexane	Rhizome	C_31_H_50_O_2_	​	Sakai et al. (2018)
38	arundoin	Hexane/Benzene	Rhizome	C_31_H_52_O	4,555–56-0	(Nishimoto et al., 1968)
39	fern-9 (11)-en-3-one	n-hexane	Rhizome	C_30_H_48_O	6,090–29-5	Sakai et al. (2023)
40	fernenol	Hexane/Benzene	Rhizome	C_30_H_50_O	4,966–00-1	(Nishimoto et al., 1968)
41	lupeol	n-hexane	Rhizome	C_30_H_50_O	545–47-1	Sakai et al. (2023)
42	*α*-amyrenone	n-hexane	Rhizome	C_30_H_48_O	638–96-0	Sakai et al. (2023)
43	*α*-amyrin	n-hexane	Rhizome	C_30_H_50_O	638–95-9	Sakai et al. (2023)
44	*β*-amyrenone	n-hexane	Rhizome	C_30_H_48_O	638–97-1	Sakai et al. (2018)
45	*β*-amyrin	n-hexane	Rhizome	C_30_H_50_O	559–70-6	Sakai et al. (2023)
46	glutinol	n-hexane	Rhizome	C_30_H_50_O	545–24-4	Sakai et al. (2023)
47	glutinone	70%EtOH	Rhizome	C_30_H_48_O	508–09-8	Liu et al. (2012)
48	friedelin	95%EtOH	Rhizome	C_30_H_50_O	559–74-0	Fu et al. (2010)
49	impallidin	n-hexane	Rhizome	C_31_H_50_O	​	Sakai et al. (2023)
50	impallidol	n-hexane	Rhizome	C_30_H_50_O	​	Sakai et al. (2023)
51	tetranorimpallidin aldehyde	n-hexane	Rhizome	C_27_H_44_O_2_	​	Sakai et al. (2023)
52	trisnorimpallidin aldehyde	n-hexane	Rhizome	C_28_H_46_O_2_	​	Sakai et al. (2023)
53	impallidin ozonide	n-hexane	Rhizome	C_31_H_52_O_4_	​	Sakai et al. (2023)
54	cycloartenol	n-hexane	Rhizome	C_30_H_50_O	469–38-5	Sakai et al. (2023)
55	4-hydroxy-cinnamic acid	70%EtOH	Rhizome	C_9_H_8_O_3_	7,400–08-0	Liu et al. (2013a)
56	caffeic acid	70%EtOH	Rhizome	C_9_H_8_O_4_	331–39-5	Jiang (2021)
57	coniferaldehyde	70%EtOH	Rhizome	C_10_H_10_O_3_	458–36-6	Jiang (2021)
58	ferulic acid	70%EtOH	Rhizome	C_10_H_10_O_4_	1,135–24-6	Jiang (2021)
59	ethyl caffeate	70%EtOH	Rhizome	C_11_H_12_O_4_	102–37-4	Jiang (2021)
60	1-O-*p*-coumaroylglycerol	70%EtOH	Rhizome	C_12_H_14_O_5_	63,529–09-9	Liu et al. (2012)
61	imperphenol B	70%EtOH	Rhizome	C_13_H_16_O_7_	​	Jiang (2021)
62	imperphenol C	70%EtOH	Rhizome	C_13_H_14_O_7_	​	Jiang (2021)
63	4-O-caffeoylquinic acid	70%EtOH	Rhizome	C_16_H_18_O_9_	905–99-7	Jiang (2021), Jiang et al. (2022)
64	(−)-4-O-feruloylquinic acid methyl ester	70%EtOH	Rhizome	C_18_H_22_O_9_	195,723–10-5	Jiang (2021), Jiang et al. (2022)
65	chlorogenic acid	70%EtOH	Rhizome	C_16_H_18_O_9_	327–97-9	Jiang (2021), Jiang et al. (2022)
66	3-O-caffeoylquinic acid methyl ester	70%EtOH	Rhizome	C_17_H_20_O_9_	123483-19-2	Jiang (2021), Jiang et al. (2022)
67	5-O-feruloylquinic acid	70%EtOH	Rhizome	C_17_H_20_O_9_	40,242–06-6	Jiang (2021), Jiang et al. (2022)
68	(1*R*,2*R*)-1-(3,4,5-trimethoxyphenyl)-1,2,3-propanetriol	70%EtOH	Rhizome	C_12_H_18_O_6_	674,359–59-2	Liu et al. (2012)
69	imperphenoside E	70%EtOH	Rhizome	C_18_H_28_O_11_	​	Jiang (2021)
70	imperphenoside D	70%EtOH	Rhizome	C_18_H_28_O_11_	​	Jiang (2021)
71	(+)-(7*S*,8*S*)-guaiacylglycerol	70%EtOH	Rhizome	C_10_H_14_O_5_	84,799–27-9	Jiang (2021)
72	(7*S*,8*S*)-syringoylglycerol	70%EtOH	Rhizome	C_11_H_16_O_6_	​	Jiang (2021)
73	(+)-(7*S*,8*S*)-guaiacylglycerol-8-O-*β*-D-glucopyranoside	70%EtOH	Rhizome	C_16_H_24_O_10_	109,280–40-2	Jiang (2021)
74	2′-O-(E)-*p*-coumaroylsalicin	70%EtOH	Rhizome	C_22_H_24_O_9_	1,257,390–08-1	Jiang (2021)
75	(1*R*,2*S*)-2-hydroxycyclohexyl-2′-O-*trans-p*-coumaroyl-*β*-D-glucopyranoside	70%EtOH	Rhizome	C_21_H_28_O_9_	15,732–48-6	Jiang (2021), Jiang et al. (2022)
76	(7*R*,8*R*)-4,7,9,9′-tetrahydroxy-3,3′-dimethoxy-8–4′-oxyneolignan-7-O-*β*-D-glucopyranoside	70%EtOH	Rhizome	C_26_H_36_O_13_	1,206,731–40-9	Ma et al. (2018)
77	(7*R*,8*S*)-4,7,9,9′-tetrahydroxy-3,3′-dimethoxy-8–4′-oxyneolignan-7-O-*β*-D-glucopyranoside	70%EtOH	Rhizome	C_26_H_36_O_13_	1,206,731–41-0	Ma et al. (2018)
78	(−)-(7*R*,8*R*)-*threo*-4,7,9,9′-tetrahydroxy-3,5,2′-trimethoxy-8-O-4′-neolignan 7-O-*β*-D-glucopyranoside	70%EtOH	Rhizome	C_27_H_38_O_13_	​	Ma et al. (2018)
79	1,2′,4′,6′-tetraacetyl-3,6-diferuloyl-sucrose	70%EtOH	Rhizome	C_40_H_46_O_21_	173,614–59-0	Jiang (2021)
80	imperphenoside F	70%EtOH	Rhizome	C_33_H_44_O_19_	​	Jiang (2021)
81	(6*R*,7*S*,8*S*)-7*α*-[(*β*-glucopyranosyl)oxy]lyoniresinol	70%EtOH	Rhizome	C_28_H_38_O_13_	873,077–48-6	Jiang (2021)
82	impecylenolide	70%EtOH	Rhizome	C_20_H_20_O_7_	1,622,990–05-9	Liu et al. (2014)
83	deacetylimpecyloside	70%EtOH	Rhizome	C_32_H_38_O_17_	1,622,990–04-8	Liu et al. (2014)
84	impecyloside	80%MeOH	Rhizome	C_34_H_40_O_18_	1,070,967–17-7	DY et al. (2008)
85	sucrose diester of 4,4′-dihydroxy-3,3′-dimethoxy-*β*-truxinic acid	70%EtOH	Rhizome	C_32_H_38_O_17_	​	Jiang (2021)
86	imperlignanoside D	70%EtOH	Rhizome	C_34_H_40_O_18_	​	Jiang (2021)
87	(−)-syringaresinol-4-O-*β*-D-glucopyranoside	70%EtOH	Rhizome	C_28_H_36_O_13_	137,038–13-2	Jiang (2021)
88	graminone A	MeOH/H_2_O	Rhizome	C_20_H_20_O_7_	161,407–72-3	Matsunaga et al. (1994b)
89	graminone B	MeOH/H_2_O	Rhizome	C_21_H_22_O_8_	161,407–73-4	Matsunaga et al. (1994b)
90	imperlignanoside E	70%EtOH	Rhizome	C_25_H_30_O_12_	​	Jiang (2021)
91	imperlignanoside C	70%EtOH	Rhizome	C_31_H_42_O_15_	​	Jiang (2021)
92	imperlignanoside B	70%EtOH	Rhizome	C_31_H_42_O_15_	​	Jiang (2021)
93	imperanene	MeOH/H_2_O	Rhizome	C_19_H_22_O_5_	163,634–08-0	Kimihiro Matsunaga (1995)
94	imperlignanoside A	70%EtOH	Rhizome	C_31_H_42_O_14_	​	Jiang (2021)
95	7-hydroxy-4-methoxy-5- methylcoumarin	70%EtOH	Rhizome	C_11_H_10_O_4_	41,680–12-0	Liu et al. (2013a)
96	siderin	70%EtOH	Rhizome	C_12_H_12_O_4_	53,377–54-1	Liu et al. (2013a)
97	4-methoxy-5-methylcoumarin-7-O-*β*-D-glucopyranoside	70%EtOH	Rhizome	C_17_H_20_O_9_	41,680–13-1	Liu et al. (2013a)
98	5-methyl coumarilic acid methyl ester 3-O-*β*-D-glucopyranoside	70%EtOH	Rhizome	C_17_H_20_O_9_	​	Ma et al. (2018)
99	5-methyl coumarilic acid methyl ester 3-O-*α*-L-rhamnopyranosyl-(1→6)-*β*-D-glucopyranoside	70%EtOH	Rhizome	C_23_H_30_O_13_	​	Ma et al. (2018)
100	4-hydroxybenzenecarboxylic acid	70%EtOH	Rhizome	C_7_H_6_O_3_	99–96-7	Liu et al. (2012)
101	3,4-dihydroxybenzoic acid	70%EtOH	Rhizome	C_7_H_6_O_4_	99–50-3	Liu et al. (2012)
102	vanillic acid	70%EtOH	Rhizome	C_8_H_8_O_4_	121–34-6	Liu et al. (2012)
103	syringic acid	70%EtOH	Rhizome	C_9_H_10_O_5_	530–57-4	Jiang (2021)
104	glucosyringic acid	70%EtOH	Rhizome	C_15_H_20_O_10_	33,228–65-8	Jiang (2021)
105	cylindol A	MeOH/H_2_O	Rhizome	C_16_H_14_O_7_	159,225–89-5	Matsunaga et al. (1994a)
106	cylindol B	MeOH/H_2_O	Rhizome	C_16_H_14_O_7_	159,225–90-8	Matsunaga et al. (1994a)
107	dimethyl 4,4′-dimethoxy-5,6,5′,6′-dimethylene-dioxybiphenyl-2,2′-dicarboxylate	95%EtOH	Rhizome	C_20_H_18_O_10_	73,536–69-3	Wang et al. (1996)
108	glycerol-1-O-*α*-D-glucuronide 3-O-benzoyl ester	70%EtOH	Rhizome	C_16_H_20_O_10_	2,280,871–15-8	Jiang (2021), Jiang et al. (2022)
109	ethylparaben	95%EtOH	Rhizome	C_9_H_10_O_3_	120–47-8	Fu et al. (2010)
110	salireposide	70%EtOH	Rhizome	C_20_H_22_O_9_	16,955–55-8	Jiang (2021)
111	4′-hydroxybenzyl-2-hydroxybenzoate-1′-O-*β*-D-glucopyranoside	70%EtOH	Rhizome	C_20_H_22_O_12_	2,365,394–81-4	Jiang (2021)
112	isovanillin	MeOH	Aerial parts	C_8_H_8_O_3_	621–59-0	Razafindrakoto et al. (2025)
113	4-hydroxy-3,5-dimethoxybenzaldehyde	70%EtOH	Rhizome	C_9_H_10_O_4_	134–96-3	Jiang (2021)
114	4-hydroxybenzaldehyde	70%EtOH	Rhizome	C_7_H_6_O_2_	123–08-0	Liu et al. (2013a)
115	3,4-dimethoxyphenyl-*β*-D-glucopyranoside	70%EtOH	Rhizome	C_14_H_20_O_8_	84,812–00-0	Jiang (2021)
116	4-hydroxy-3-methoxyphenyl-*β*-D-xylopyranosyl (1→6)-O-*β*-D-glucopyranoside	70%EtOH	Rhizome	C_18_H_26_O_12_	619,319–71-0	Jiang (2021)
117	cuneataside D	70%EtOH	Rhizome	C_19_H_28_O_12_	871,720–19-3	Jiang (2021)
118	3,4-dimethoxyphenyl-(6-O-*α-*L-rhamnopyranosyl)-*β*-D-glucopyranoside	70%EtOH	Rhizome	C_20_H_30_O_12_	872,885–48-8	Liu et al. (2013a)
119	3,4-dimethoxyphenyl-1-O-*β*-D-apiofuranosyl-(1→6)-*β*-D-glupyranoside	70%EtOH	Rhizome	C_19_H_28_O_12_	761,446–98-4	Jiang (2021)
120	3,4,5-trimethoxyphenyl 1-O-*β*-apiofuranosyl (1''→6′)-*β*-glucopyranoside	70%EtOH	Rhizome	C_20_H_30_O_13_	87,562–76-3	Jiang (2021)
121	salicin	70%EtOH	Rhizome	C_13_H_18_O_7_	138–52-3	Liu et al. (2012)
122	echipuroside A	70%EtOH	Rhizome	C_20_H_30_O_11_	445,486–55-5	Jiang (2021)
123	poliothrysoside	70%EtOH	Rhizome	C_20_H_22_O_9_	18,463–25-7	Jiang (2021)
124	imperphenoside A	70%EtOH	Rhizome	C_20_H_28_O_12_	​	Jiang (2021)
125	seguinoside K 4-methylether	70%EtOH	Rhizome	C_27_H_34_O_15_	1,588,477–87-5	Liu et al. (2014)
126	seguinoside K	70%EtOH	Rhizome	C_26_H_32_O_15_	257,888–26-9	Liu et al. (2014)
127	11,16-dihydroxypregn-4-ene-3,20-dione	Ethyl acetate	Aerial parts	C_21_H_30_O_4_	3,034,577–60-8	Wang et al. (2018)
128	2-methoxyestrone	Ethyl acetate	Aerial parts	C_19_H_24_O_3_	362–08-3	Wang et al. (2018)
129	stigmast-4-en-3-one	MeOH	Aerial parts	C_29_H_48_O	1,058–61-3	Razafindrakoto et al. (2025)
130	sitosterol	95%EtOH	Rhizome	C_29_H_50_O	83–46-5	Fu et al. (2010)
131	daucosterol	70%MeOH	Aerial parts	C_35_H_60_O_6_	474–58-8	Mohamed et al. (2009)
132	*β*-sitosterol-3-O-*β*-D-glucopyranosyl-6′-tetradecanoate	70%MeOH	Aerial parts	C_49_H_86_O_7_	120,000–27-3	Mohamed et al. (2009)
133	cylindrene	MeOH/H_2_O	Rhizome	C_15_H_20_O_2_	158,204–49-0	Matsunaga et al. (1994c)
134	impecylone	70%EtOH	Rhizome	C_14_H_14_O_4_	1,622,990–03-7	Liu et al. (2014)
135	cylindracid B	MeOH	Plant	C_11_H_16_O	​	Nago et al. (2021)
136	cylindracid A	MeOH	Plant	C_15_H_22_O_4_	​	Nago et al. (2021)
137	(6*S*,9*S*)-roseoside	70%EtOH	Rhizome	C_19_H_30_O_8_	185,414–25-9	Jiang (2021), Jiang et al. (2022)
138	(6*R*,9*S*)-13-nor-5-carboxyblumenol C 9-O-*β*-glucoside	70%EtOH	Rhizome	C_19_H_30_O_9_	906,646–94-4	Jiang (2021), Jiang et al. (2022)
139	5-hydroxymethylfurfural	95%EtOH	Rhizome	C_6_H_6_O_3_	67–47-0	Fu et al. (2010)
140	3,4-dihydroxybutyric acid	70%EtOH	Rhizome	C_4_H_8_O_4_	1,518–61-2	Liu et al. (2012)
141	palmitic acid	95%EtOH	Rhizome	C_16_H_32_O_2_	57–10-3	Wang et al. (1996)

### 5.2 Triterpenoids

Triterpenoids are also a major metabolite in *I. cylindrica*, with 26 types isolated in total. Most of them are pentacyclic triterpenoids, tetracyclic triterpenes are in the minority. Notably, rare E:B-friedo-hopane type triterpenoids (29–30 in Figure 2; Table 2) are discovered, besides, five novel skeletal triterpenoids (49–53) have been isolated from *I. cylindrica*, which belong to relatively rare triterpenoid types.

### 5.3 Phenylpropanoids

Phenylpropanoid metabolites isolated from *I. cylindrica* are classified into three categories: simple phenylpropanoids, lignans, and coumarins. Simple phenylpropanoids include common phenylpropionic acids such as caffeic acid, ferulic acid, and chlorogenic acid, along with some aldehydes, alcohols, and glycoside compounds. Lignans are a class of natural products formed by the oxidative polymerization of phenylpropanoids, and a diverse array of lignans have been isolated from *I. cylindrica*: common types include oxo-neolignans (76–78 in Figure 2; Table 2); some belong to the dibenzylbutyrolactone type (82, 90); and others are norlignan metabolites (91–94). Several metabolites exhibit unique structures, for instance, *β*-truxinic acid derivatives (83–86), which serve as crucial lead compounds for the development of novel anti-inflammatory drugs (Chi et al., 2006); additionally, certain metabolites (87–89) possess a fused tetrahydrofuran structure. A total of five coumarin metabolites have been isolated from this plant.

### 5.4 Aromatics

A total of 27 simple aromatic metabolites have been isolated from *I. cylindrica*, including aromatic acids (100–111 in Figure 2; Table 2), aromatic aldehydes (112–114), phenolic acids (115–126). In addition, there are various phenolic acid glycosides with acids or aldehydes as aglycones, linked to sugars such as glucose, glucuronic acid, and rhamnose.

### 5.5 Other metabolites

Steroidal metabolites isolated from *I. cylindrica* are relatively scarce, with only six metabolites, namely, 11,16-dihydroxypregn-4-ene-3,20-dione (127 in Figure 2; Table 2), 2-methoxyestrone (128), stigmast-4-en-3-one (129), sitosterol (130), daucosterol (131) and *β*-sitosterol-3-O-*β*-D-glucopyranosyl-6′-tetradecanoate (132). In addition, several other metabolites have been isolated from *I. cylindrica*, such as sesquiterpenes (133,136). This also includes metabolites like 5-hydroxymethylfurfural (139), 3,4-dihydroxybutyric acid (140), and palmitic acid (141).

## 6 Pharmacology

**FIGURE 3:**
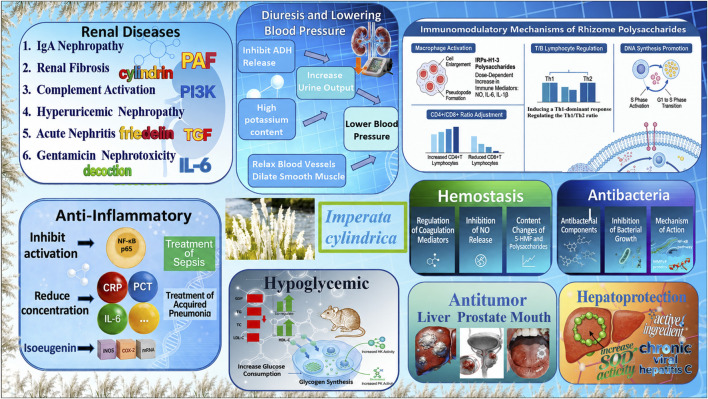
Overview of the pharmacological effects of *Imperata cylindrica*.

Existing pharmacological studies suggest that *I. cylindrica* exhibits anti-inflammatory, antioxidant, immunomodulatory, renal protective, hepatoprotective, hypoglycemic, hemostatic, and diuretic activities. However, literature analysis reveals numerous limitations in existing pharmacological studies on *I. cylindrica*. The majority of current investigations focus on crude extracts, while only a small number of isolated metabolites have been subjected to activity tests. Pharmacological data for most monomeric metabolites remain absent, which makes it impossible to identify the core bioactive substances responsible for the plant’s pharmacological effects and establish efficacy-related quality control criteria. There are very few human clinical studies, and large-sample, multi-center, high-quality randomized controlled trials are lacking. Accordingly, high-level evidence to support its clinical application is insufficient, and the long-term safety and efficacy of *I. cylindrica* in humans have not been fully verified. Although included animal studies adopted standardized disease models and administration regimens, notable methodological heterogeneity exists due to inconsistent animal species, dosages, administration routes and pharmacodynamic evaluation endpoints. Most *in vitro* studies merely examined the impacts of extracts or single metabolites on cellular inflammatory factors, cell proliferation and apoptosis. Only a few works have clarified specific signaling pathways. In-depth exploration of drug targets, structure-activity relationships and upstream and downstream regulatory networks is still inadequate, leaving the molecular mechanisms underlying the pharmacological activities of *I. cylindrica* incompletely elucidated. In addition, the diverse phytochemicals in *I. cylindrica* may cause non-specific interference in biological assays, which has not been addressed in previous work. To systematically organize these reported activities and facilitate intuitive access for readers, the key pharmacological findings of *I. cylindrica* are summarized in (Table 3) and pictured in (Figure 3).

**TABLE 3:** Summary of pharmacological activities of *Imperata cylindrica*.

Activity	Extract/Metabolite	Model	Dose	Mechanism/Key findings	References
Anti-inflammatory	Aqueous decoction	Xylene-induced ear edema mice Carrageenan/zymosan-A paw swelling rats; Acetic-acid capillary permeability mice Nystatin-induced paw edema mice (*in vivo*)	2.5/5.0 g/kg	inhibiting inflammatory mediators; decreasing ear swelling, paw swelling, and vascular permeability no significant effect (nystatin-induced)	Yue et al. (2006)
Anti-inflammatory and antioxidant	Ethanol extract of rhizome	LPS-induced sepsis mice model (*in vivo*)	90/115 mg/kg	90 mg/kg: increasing GPx3, decreased monocytes, decreasing PMN infiltration, decreased TNF-α, decreased FABP4 115 mg/kg: increasing platelets and hepatocytes, decreasing lymphocytes and FABP4, leading to improved survival	Putri et al. (2021)
Anti-inflammatory	*Imperata cylindrica* polysaccharide	CLP-induced sepsis rat model (*in vivo*)	10 mg/kg/d (low), 20 mg/kg/d (high)	reducing NF-κB p65 expression; alleviating alveolar-capillary barrier damage; dose-dependent protection	Jiang and Yu (2016)
Anti-inflammatory	Aqueous decoction	Acquired pneumonia patients (clinical)	200 mL, 2 doses per day	Decreasing CRP, PCT, IL-6, IL-8, and TNF-α via inhibition of the TLR4/NF-κB pathway	Li et al. (2021)
Anti-inflammatory	Isoeugenin (metabolite 28)	LPS-induced RAW 264.7 macrophages (*in vitro*)	15/25/50 μg/ml	The IC_50_ on the inhibition of NO formation:9.33 μg/mL; decreasing iNOS, COX-2, and reducing TNF-α, IL-6 and IL-1β mRNA expression	An et al. (2015)
Antioxidant, Analgesic, Anti-inflammatory, Antipyretic	Antioxidant, Analgesic, Anti-inflammatory, Antipyretic	*In vitro* and *in vivo* mice models (*in vivo*)	*in vivo*: 50–200 mg/kg	IC_50_ = 192.07 μg/mL (DPPH); Scavenging DPPH radicals and reducing Fe^3+^ *in vitro*; inhibiting writhing, carrageenan edema, and LPS-induced fever *in vivo*; no toxicity observed in acute and sub-chronic studies	Rinah et al. (2021)
Renal protection	Aqueous decoction	IgA nephropathy rat model (*in vivo*)	10 g/kg/d	*I. cylindrica* and its compound decoction can significantly reduce hematuria, proteinuria of the IgA nephropathy rats and reduce the pathological changes and improve renal function	Yin et al. (2011)
Anti-renal fibrosis	Cylindrin (metabolite 34)	FA-induced mouse renal fibrosis (*in vivo*)	120 mg/kg/d (high), 60 mg/kg/d (low)	Inhibiting M2 macrophage polarization via the LXRα/PI3K/AKT pathway	Li et al. (2023)
Hyperuricemic nephropathy	*Imperata cylindrica* polysaccharide	Adenine and potassium oxonate-induced hyperuricemic nephropathy mice (*in vivo*)	600 mg/kg/d	reducing intestinal damage, decreasing the expression of inflammatory factors, maintaining the integrity of the intestinal barrier, inhibiting the growth of harmful bacteria	Yu et al. (2024)
Diuretic	Fresh rhizome of *Imperata cylindrica* freeze-dried, sun-dried	Rat water-salt load model (*in vivo*)	fresh rhizome:10/5 g/kg freeze-dried:31.1/15.5 g/kg sun-dried:8.84/4.42 g/kg	influencing hypothalamic ADH release	Zhang (2022)
No diuretic effect	Aqueous decoction	Healthy human volunteers (18–27 years), placebo-controlled, double-blind crossover (clinical)	0.6 L/d	No significant difference in 24 h or 12 h urine output vs. placebo; no change in Na^+^/K^+^ excretion; no pure diuretic effect detected	Dat et al. (1992)
Antihypertensive	Ethanolic extract of leaves	Cat (anesthetized) (*in vivo*)and Rabbit jejunum (*in vitro*)	10–320 mg/ml	Heart pressure in cats: 266 to 180 mmHg at 160–320 mg/ml; amplitude of smooth muscle contraction in rabbit jejunum reduced in dose-dependent manner vs. adrenaline control	Mak-Mensah et al. (2010)
Hemostasis	Carbonized the rhizome of *I*. *cylindrica*, tannin fraction, ethyl acetate fraction	SD rats (*in vivo*)	2 ml/100 g/d	Shortened prothrombin time, thrombin time, activated partial thromboplastin time, increased fibrinogen content, promoted platelet aggregation, and had no effect on	Xu et al. (2010)
Hemostasis	Aqueous extract	RAW 264.7 macrophages (*in vitro*)	0.125, 0.25, 0.5 g crude drug/L	Significantly inhibiting lipopolysaccharide-induced nitric oxide production in macrophages	Liao et al. (2007)
Antitumor	Aqueous extract	Human hepatoma cell line SMMC-7721 (*in vitro*)	2.5–40 mg/mL	Inhibiting proliferation in time- and dose-dependent manner; inducing apoptosis; arresting cell cycle in S phase and decreasing the proportion of G2/M phase cells	Bao et al. (2013)
Antitumor	Aqueous extract	Mouse hepatoma H22 cell xenograft model (*in vivo*)	40.909 mg/mL, 0.5 mL, twice daily	Significantly inhibiting the growth of mouse hepatoma xenograft, with an inhibition rate of 33.85%	Sun et al. (2015)
Antitumor	Arundoin (metabolite 38)	PC3 human prostate cancer cells (*in vitro*)	20, 40, 80 μmol/L	Inhibiting cell proliferation in time and dose dependent manner; inducing apoptosis; arresting cell cycle in G0/G1 phase; activating poly ADP ribose polymerase and regulating apoptosis-related proteins	Chen (2017)
Antitumor	Methanol extract of leaves	Human oral squamous carcinoma SCC-9 cells *(in vitro*)	10–640 μg/mL	Inhibiting cell proliferation in dose dependent manner; reducing clonogenic ability; arresting cell cycle in G2/M phase; inducing apoptosis through DNA fragmentation	Keshava et al. (2016), Keshava et al. (2020)
Immunomodulation	*Imperata cylindrica* polysaccharide	RAW 264.7 mouse macrophages (*in vitro*)	50–800 μg/mL	Promoting macrophage proliferation; enhancing phagocytic activity; inducing morphological activation; increasing the production of nitric oxide, tumor necrosis factor-α, interleukin-6 and reactive oxygen species	Sun et al. (2025)
Immunomodulation	Aqueous decoction	Normal mice and immunosuppressed mice induced by hydrocortisone (*in vivo*)	1.0 g/mL	Increasing the percentage of ANAE-positive lymphocytes; raising the percentage of CD4^+^ T lymphocytes; reducing the percentage of CD8^+^ T lymphocytes; regulating the CD4+/CD8+ ratio to normal levels	Fu et al. (2000)
Immunomodulation	Aqueous decoction	Mice (*in vivo*)	5 g/kg, 10 g/kg	Enhancing the phagocytic rate and phagocytic index of peritoneal macrophages; increasing the percentage of T helper cells; promoting the production of interleukin-2; showing no significant effect on humoral	Lu and Huang (1996)
Antibacterial	Aqueous decoction, 50% ethanol extract, ethyl acetate extract, acetone extract of the rhizome of *Imperata cylindrica*	*Enterobacter aerogenes, Escherichia coli, Staphylococcus aureus, Saccharomyces albicans, Bacillus subtilis* (*in vitro*)	1 g/mL	Inhibiting the growth of five species; showing the strongest inhibitory effect on *Escherichia coli*; reducing the minimum inhibitory concentration against different test strains	Li and Zhang (2012)
Antibacterial	Cylindricines A and B, cylindracid B (metabolite 17, 18, 135)	*Escherichia coli, Enterobacter aerogenes, Staphylococcus aureus* (*in vitro*)	16–512 μg/mL	Inhibiting the growth of multidrug-resistant gram-positive and gram-negative bacteria; showing potent inhibitory activity against *Staphylococcus aureus*	Nago et al. (2021)
Antibacterial, and inflammatory	“Baimao-Longdan-Congrong-Fang”	*Staphylococcus aureus* (*in vitro*), mice model of methicillin-resistant *Staphylococcus aureus* pneumonia (*in vivo*)	1.85–7.40 g/kg	Inhibiting the activity of sortase A; reducing the expression of alpha-hemolysin; regulating the tumor necrosis factor-α/tumor necrosis factor receptor 1/nuclear factor-κB/matrix metalloproteinase 9 axis; alleviating lung injury; decreasing lung bacterial load	Jiang et al. (2025)
Hepatoprotection	Aqueous decoction	Mice with alcoholism-induced liver and brain injury (*in vivo*)	12 g/kg	Enhancing the activity of superoxide dismutase; reducing the content of malondialdehyde; improving the ability to scavenge hydroxyl radicals; protecting against alcohol-induced liver and brain injury	Lan et al. (2012)
Hepatoprotection	metabolite 77,98, 99	Human hepatocellular carcinoma HepG2 cells (*in vitro*)	10 μmol/L	Alleviating N-acetyl-*p*-aminophenol-induced HepG2 cell injury; showing significant hepatoprotective activity	Ma et al. (2018)
Hypoglycemic	*Imperata cylindrica* polysaccharide	mice with streptozotocin-induced diabetes mellitus (*in vivo*)	100/200 mg/kg	Regulating glucose and lipid metabolism disorders; improving glucose tolerance; increasing liver glycogen content; reducing blood glucose and blood lipid levels	Cui et al. (2012)
Hypoglycemic	Extract of rhizome	SD rats with periodontitis (*in vivo*)	0.5%, 1.5%, 3.0% added in toothpaste	Inhibiting six periodontal pathogens; reducing inflammatory factors; improving periodontal health; showing no oral toxicity	Wei et al. (2024)

### 6.1 Anti-inflammatory

Inflammation is closely associated with multiple pathological processes. Natural products with favorable safety profiles represent promising sources for anti-inflammatory agents. *Imperata cylindrica* has long been used to treat inflammatory conditions, and modern studies have validated its anti-inflammatory effects via regulation of key inflammatory mediators and signaling pathways.

The 5.0 g/kg aqueous decoction of the rhizome of *I. cylindrica* significantly inhibits xylene-induced ear edema in mice. Separately, the rhizome’s aqueous decoction reduces acetic acid-induced increase in peritoneal capillary permeability in mice and counteracts carrageenan/zymosan A-induced rat paw swelling, showing a dose-dependent relationship, which may involve the inhibition of multiple inflammatory mediators (Yue et al., 2006). In a septic mouse model, *I. cylindrica* ethanol extract (ECGR) at doses of 90 mg/kg body weight (BW) and 115 mg/kg BW showed a positive alleviating effect on the severe condition. Specifically, the 90 mg/kg BW dose significantly increased glutathione peroxidase 3 (GPx3) activity, reduced monocyte count, polymorphonuclear cell infiltration in the liver and heart, TNF-α expression, and fatty acid binding protein 4 (FABP4) levels; the 115 mg/kg BW dose notably elevated platelet levels, hepatocyte count, and cardiomyocyte count, while decreasing lymphocyte count, monocyte count, spleen white pulp length and width, and FABP4 levels, thereby improving sepsis (Putri et al., 2021). In model-induced septic rats, nuclear factor-κB (NF-κB) p65 was widely expressed in infiltrating inflammatory cells, alveolar epithelial cells, vascular endothelial cells, and airway mucosal epithelial cells. After intervention with *I. cylindrica* lipopolysaccharide at doses of 10 mg/kg·d^−1^ (low dose) and 20 mg/kg·d^−1^ (high dose) via intragastric administration for 7 consecutive days, the nuclear expression of nuclear factor-κB (NF-κB) p65 showed a significant dose-dependent downregulation. It is speculated that the therapeutic effect of *I. cylindrica* lipopolysaccharide on sepsis-induced acute lung injury may be achieved by inhibiting the activation and nuclear translocation of NF-κB p65, thereby reducing the release of multiple inflammatory mediators (e.g., TNF-α, IL-8) and alleviating the systemic inflammatory response (Jiang and Yu, 2016). Additionally, in the treatment of acquired pneumonia, it can reduce serum levels of C-Reactive Protein (CRP), Procalcitonin (PCT), IL-6, IL-8, and TNF-α. The mechanism is related to inhibiting the activation of the TLR4/NF-κB signaling pathway, achieving downregulation of inflammatory factor release and improving clinical symptoms (Li et al., 2021).

*I. cylindrica* contains flavonoids and isoeugenol metabolites with anti-inflammatory effects. Among them, isoeugenin isolated from its rhizome significantly inhibited the mRNA expression levels of inducible nitric oxide synthase (iNOS), cyclooxygenase-2 (COX-2), and pro-inflammatory cytokines in macrophage RAW 264.7 cells. In summary, these results indicate that the anti-inflammatory activity of isoeugenol is related to the downregulation of iNOS, COX-2, and pro-inflammatory cytokines, thereby exhibiting anti-inflammatory activity (An et al., 2015). In addition, the methanol extract of *I. cylindrica* (MEIC) not only exhibits significant *in vitro* antioxidant properties but also exerts dose-dependent analgesic, anti-inflammatory, and antipyretic effects *in vivo.* Furthermore, MEIC has not shown any toxicity, which confirms the safety of this plant (Rinah et al., 2021).

### 6.2 The treatment of renal diseases

IgA nephropathy is the most prevalent primary glomerulonephritis and a major cause of renal dysfunction. It involves IgA deposition, inflammatory mediator release, and progressive renal fibrosis (Guizhen et al., 2021). *I. cylindrica* has been shown to ameliorate IgA nephropathy by reducing hematuria, proteinuria, and renal injury (Yin et al., 2011).

In the treatment of renal fibrosis, cylindrin isolated from *I. cylindrica* alleviates M2 macrophage polarization by inhibiting the liver receptor α (LXRα) and phosphatidylinositol 3-kinase (PI3K)/protein kinase B (AKT) signaling pathways, thereby relieving renal fibrosis (Li et al., 2023). Additionally, friedelin, trans-*p*-coumaric acid, and 4-hydroxy-3-methoxybenzoic acid isolated from this botanical drug can all ameliorate excessive complement activation in animal models of membranoproliferative glomerulonephritis, exerting protective effects on the kidneys (Fu et al., 2010). Treatment of hyperuricemic nephropathy (HN) mice with polysaccharide extract of *I. cylindrica* can effectively reduce the overexpression of pro-inflammatory cytokines such as IL-6, IL-17, and IFN-γ, and also regulate the abundance of gut microbiota and their metabolites, thus exerting multi-pathway and multi-target therapeutic effects on HN (Yu et al., 2024). Satisfactory curative effects have also been achieved in treating acute diseases. The application of compound rhizome decoction has shown favorable therapeutic efficacy in patients with acute nephritis. Overall, *I. cylindrica* exerts comprehensive protective and therapeutic effects on the kidneys. Studies have confirmed that its ethanol extract exerts significant renoprotective effects against gentamicin-induced nephrotoxicity, effectively improving levels of creatinine and urea as well as related hematological parameters. In summary, *I. cylindrica* exhibits consistent renal protective effects across multiple kidney disease models, including IgA nephropathy, renal fibrosis, membranoproliferative glomerulonephritis, and hyperuricemic nephropathy. Active metabolites such as cylindrin and friedelin suppress complement overactivation, macrophage polarization, and fibrotic signaling.

### 6.3 Diuresis and lowering blood pressure

As a traditional medicinal plant, *I. cylindrica* holds significant pharmacological value in regulating body fluid metabolism and protecting cardiovascular health, with its prominent diuretic and hypotensive activities supported by a series of experimental and clinical studies A study using a rat model with water and salt load demonstrated that all tested preparations (fresh, sun-dried, and freeze-dried) increased both urine output and urinary ion excretion. Compared with the control group, all administration groups showed decreased levels of antidiuretic hormone (ADH), suggesting that *I. cylindrica* regulates urine volume by influencing hypothalamic ADH release. Among the various preparations, the fresh form of *I. cylindrica* exhibited superior efficacy, providing new research perspectives for this less-studied form (Zhang, 2022). Additionally, the high potassium content in this plant may also contribute to its diuretic effect (Yu et al., 1995). Notably, a rigorous double-blind, placebo-controlled crossover clinical trial in healthy humans found no significant diuretic effect of *I. cylindrica* under standardized conditions (Dat et al., 1992). However, this negative result should be interpreted with caution due to important limitations: the study was conducted in healthy volunteers rather than patients with edema or damp-heat syndrome, the dosage and extraction method followed traditional Vietnamese practice which may not be optimal, and the short intervention period may not reflect the mild, gradual, and cumulative diuretic action observed in clinical practice. Therefore, the diuretic effect of *I. cylindrica* is supported by abundant preclinical evidence but remains inconclusive in healthy humans, and further clinical studies in target patient populations are still needed.

Precisely due to its rich potassium salt content, it may also alleviate glomerular vasospasm, thereby increasing renal blood flow and glomerular filtration rate to produce a diuretic effect. Simultaneously, as renal ischemia improves, the production of renin decreases, helping to restore normal blood pressure. Experiments have proven that *I. cylindrica* hypotensive tea can markedly reduce blood pressure in rat models of hypertension (Shi et al., 2008). And its leaf extracts exert significant antihypertensive activity through the relaxation and dilation of smooth muscle in blood vessels (*in vivo*) and the gastrointestinal tract (*in vitro*) (Mak-Mensah et al., 2010). At the clinical level, it has also demonstrated mild, sustained, and safe antihypertensive effects in the treatment and prevention of mild to moderate hypertension.

### 6.4 Hemostasis

*I. cylindrica* is categorized as a blood-cooling and hemostatic agent in traditional medicine. It is commonly used in clinical practice to treat conditions such as heat strangury, blood strangury, and edema, and is particularly suitable for bleeding disorders caused by blood-heat (Wei et al., 2015). Coagulation is regulated through multiple pathways, and research indicates that charred *I. cylindrica* can influence both the intrinsic and extrinsic coagulation pathways, thereby enhancing its hemostatic effect. The tannin fraction of charred *I. cylindrica* has been shown to significantly increase thromboxane B2 (TXB2) and decrease 6-keto-PGF1α levels. These two metabolites are stable plasma metabolites of thromboxane A2 (TXA2) and prostacyclin (PGI2), respectively. TXA2 promotes platelet aggregation, while PGI2 inhibits it (Xu et al., 2010). NO is a critical mediator in the regulation of vascular tone. Inhibiting NO synthesis can restore the contractile activity of splanchnic blood vessels in animals with portal hypertension, leading to vasodilation and inhibition of platelet aggregation. By inhibiting nitric oxide (NO) production, its rhizome extract may mediate part of its hemostatic action (Liao et al., 2007).In fact, the crude extract of rhizome also exhibits certain hemostatic activity, though it is less effective than its charred counterpart (Song and Chen, 2000). Studies have confirmed that the content of 5-hydroxymethylfurfural (5-HMF), a hexose degradation product, increases significantly after charring, while polysaccharide content decreases. This chemical transformation is likely responsible for the enhanced coagulation activity of charred *I. cylindrica* (Zhang, 2021).

### 6.5 Antitumor

Globally, cancer is a disease that severely affects human populations. Scientific research interest is shifting toward naturally derived metabolites, as they are considered to have fewer toxic side effects compared to current treatments such as chemotherapy (Greenwell and Rahman, 2015). Its rhizome extract has demonstrated promising anticancer activity, showing significant inhibitory effects on the proliferation of various tumor cell lines, promoting apoptosis, altering the cell cycle, and exhibiting no toxic side effects on normal cells (Keshava et al., 2020). The following sections provide a separate review of *I. cylindrica*’s effects on cancers of different organs.

Liver cancer, as a malignant tumor that seriously endangers the health and lives of the Chinese population, presents alarming incidence and mortality rates. According to data released by the China National Cancer Center, in 2022, the number of liver cancer cases in China accounted for 42.5% of global cases, ranking fourth in the number of new cancer cases and fifth in incidence rate among all types of cancer (Chinese Society of Liver Cancer, Chinese Anti-Cancer Association, 2025). In recent years, traditional Chinese medicine has made significant efforts in the prevention and treatment of liver cancer, including TCM therapies, clinical medication, and anti-cancer mechanisms. *I. cylindrica*, as one of the Chinese medicinal botanical drugs with anti-cancer activity, has been widely used in numerous formulations (Greenwell and Rahman, 2015). The “Liver Cancer Compound Formula”, an empirical formula derived from traditional Chinese medicine clinical experience and composed of four botanical drugs including Imperatae Rhizoma, Polygoni Orientalis Fructus (the fruit of *Persicaria orientalis* (L.) Spach [Polygonaceae]), Ophicalcitum (mainly composed of CaCO_3)_ and Coicis Semen (the seed kernel of *Coix lacryma-jobi* L. [Poaceae]), demonstrates significant therapeutic effects on transplanted hepatocellular carcinoma in mice with relatively low side effects (Sun et al., 2015). Another formula, the “Yunpi Qinggan Decoction” (containing Imperatae Rhizoma, Atractylodis Rhizoma (the rhizome of *Atractylodes lancea* (Thunb.) DC. [Asteraceae]), Crataegi Fructus (the fruit of *Crataegus pinnatifida* Bunge [Rosaceae]), Bupleuri Radix (the root of *Bupleurum chinense* DC. [Apiaceae] and *Bupleurum scorzonerifolium* Willd. [Apiaceae], etc.), shows remarkable efficacy in treating postoperative liver injury in primary liver cancer patients and improves their quality of life (Wang et al., 2014).

A series of studies applying modern pharmacological research methods have revealed that the aqueous extract of its rhizome significantly promotes apoptosis in the human hepatocellular carcinoma cell line SMMC-7721. This effect is achieved by inhibiting the proportion of cells in the G2/M phase and arresting the cell cycle in the S phase. Through a series of immunopharmacological experiments, including assessments of immune organ weight and organ indices, synergistic and detoxifying effects, and lymphocyte transformation tests of mouse splenic T and B cells, it has been demonstrated that its rhizome polysaccharides possess immunomodulatory functions. Furthermore, the anti-liver cancer effects of the screened active component group of *I. cylindrica* were validated *in vitro* using serum pharmacology methods, indicating its series of immunoregulatory effects, which are key factors contributing to its anti-cancer activity (Bao et al., 2013).

Prostate cancer is a common malignant tumor in men, ranking second in global incidence and fifth in mortality among male malignancies. Comprehensive treatment approaches, including surgery, endocrine therapy, radiotherapy, and traditional Chinese medicine, can help improve cure rates and quality of life (Li and Dai, 2025). Arundoin, an important triterpenoid metabolite isolated from *I. cylindrica*, has been found to significantly reduce the activity of human prostate cancer PC3 cells and induce their apoptosis. This effect is likely mediated by increasing the concentration of the active form of caspase, thereby enhancing the cleavage of its substrate PARP, which plays a critical role in apoptosis. Furthermore, arundoin upregulates the expression of the pro-apoptotic protein Bax and downregulates the expression of the anti-apoptotic protein Bcl-2. These findings further validate the apoptosis-inducing activity of arundoin in PC3 cells, providing a new rationale for the application of this plant in the treatment of prostate cancer (Chen, 2017).

Research has demonstrated that rhizome extract exhibits specific anti-cancer effects on human tongue squamous cell carcinoma (SCC-9) cells, while showing no significant impact on normal NIH/3T3 fibroblast cells. The extract effectively induced death in SCC-9 cancer cells by reducing clonogenic ability, triggering DNA fragmentation, and promoting apoptosis. These effects were further verified through the expression levels of caspase-3 and caspase-8 genes. (Keshava et al., 2016; Keshava et al., 2020).

Crude extracts and triterpenoids from *I. cylindrica* exert broad antitumor effects in liver, prostate, and oral cancer cells. Arundoin and other metabolites induce apoptosis, block cell cycle progression, and regulate Bax/Bcl-2 and caspase pathways. But all evidence remains at the *in vitro* or animal level; no clinical or translational data exist.

### 6.6 Immunological activity

Immune cells secrete immune mediators such as nitric oxide (NO), interleukin-6 (IL-6), and tumor necrosis factor-alpha (TNF-α), as well as cytokines and chemokines. These substances activate T and B lymphocytes, thereby enhancing antigen-specific immunity (Sato et al., 2015). Furthermore, they modulate the balance of T-cell subsets, particularly the Th1/Th2 ratio, which further amplifies the immune response. By preferentially inducing a Th1-dominant response, they exhibit immunomodulatory effects. Meng-Ge Sun et al. isolated three polysaccharides (IRPs-H1-3) from rhizome. Treatment of macrophages with these polysaccharides resulted in cell enlargement and pseudopodia formation, which are characteristic signs of activation. Quantitative analysis of the cell culture supernatant confirmed a dose-dependent increase in the secretion of immune mediators, including NO, IL-6, and IL-1β. Within the concentration range of 50–200 μg/mL, all three IRPs-H metabolites significantly enhanced the generation of reactive oxygen species (ROS), indicating activation at the molecular level. In summary, the three IRPs-H polysaccharide fractions demonstrate significant immunomodulatory activity through multiple mechanisms: promoting cell proliferation, enhancing phagocytosis, regulating the secretion of immune molecules, and inducing ROS generation (Sun et al., 2025).

Additionally, *I. cylindrica* significantly increases the percentage of T-helper (Th) cells and elevates IL-2 levels in mice, thereby enhancing overall immune function. Its water decoction markedly increases the percentage of ANAE-positive (Nonspecific Esterase Staining) cells and CD_4_^+^T lymphocytes in the peripheral blood of both normal and immunocompromised mice, while reducing the percentage of CD_8_^+^T lymphocytes. This adjustment shifts the CD_4_^+^T/CD_8_^+^T ratio towards normal levels, ultimately strengthening cellular immune function (Fu et al., 2000; Lu and Huang, 1996). Further research revealed that its rhizome polysaccharides primarily act on the S phase of the cell cycle. They significantly promote DNA synthesis in PHA-activated T lymphocytes, facilitating the transition from the G1 to the S phase, and thus exhibit a clear proliferative effect on normal human peripheral blood T lymphocytes (Lu et al., 2004).These findings collectively indicate that the polysaccharides from the its rhizome possess strong potential as potent immunomodulatory agents.

### 6.7 Antibacterial

*Imperata cylindrica,* traditionally valued for its “heat-clearing” property in herbal medicine, has been validated by modern pharmacological studies to possess broad-spectrum antimicrobial activity. It not only contains a variety of natural antibacterial metabolites but also exerts significant bacteriostatic effects when formulated into traditional Chinese medicine prescriptions, offering a novel perspective for addressing clinical infections and antimicrobial resistance. *I. cylindrica* extract exhibits inhibitory effects against both gram-positive and gram-negative bacteria, as well as fungi (Li and Zhang, 2012). Metabolites such as cylindricines A, cylindricines B, and cylindracid B, which were first isolated from nature from this plant, have been confirmed to possess significant activity against a variety of bacteria. This, combined with known antimicrobial metabolites like imperanene, arundoin, and α-biphenyl diester, provides a modern scientific explanation for the traditional “heat-clearing” pharmacological action of *I. cylindrica* (Nago et al., 2021).

The traditional formula “Baimao-Longdan-Congrong-Fang” (composed of Imperatae Rhizoma, Gentianae Radix et Rhizoma and Cistanches Herba), known for its heat-clearing and deficiency-tonifying effects, has demonstrated significant efficacy in inhibiting *Staphylococcus aureus*. Its mechanism of action involves regulating the TNF-α-activated NF-κB signaling pathway while downregulating the expression of MMP-9 protein (Jiang et al., 2025). Particularly in the current context of increasing antimicrobial resistance in *S. aureus*, this finding undoubtedly provides a fresh perspective for researching the mechanisms of traditional Chinese medicine formulas in treating *S. aureus* infections.

### 6.8 Hepatoprotection

Superoxide dismutase (SOD) primarily functions to catalyze the disproportionation of oxygen free radicals. The level of SOD activity can indirectly reflect the body’s capacity to scavenge these radicals. In cases of alcohol poisoning or chronic alcohol consumption, increased ethanol metabolism leads to the accumulation of oxygen free radicals in the body, resulting in a significant decrease in SOD activity in liver tissue and a weakened ability to suppress oxygen free radicals. After treatment with *I. cylindrica*, SOD activity in liver tissue is markedly enhanced, and free radical levels are significantly reduced (Lan et al., 2012). Furthermore, three matabolites isolated from *I. cylindrica*: 5-methyl coumarilic acid methyl ester 3-O-*β*-D-glucopyranoside, (7*R*,8*S*)-4,7,9,9′-tetrahydroxy-3,3′-dimethoxy-8–4′-oxyneolignan-7-O-*β*-D-glucopyranoside, and 5-methyl coumarilic acid methyl ester 3-O-*α*-L-rhamnopyranosyl-(1→6)-*β*-D-glucopyranoside, demonstrated significant hepatoprotective activity *in vitro* against N-acetyl-p-aminophenol (APAP)-induced damage in HepG2 cells (Ma et al., 2018).Furthermore, for chronic viral hepatitis C, treatment with a *I. cylindrica* decoction in combination with interferon and ribavirin demonstrates superior efficacy compared to interferon and ribavirin alone, particularly in terms of liver protection, enzyme reduction, and alleviating side effects (Qiu et al., 2012). These findings collectively indicate that it possesses notable liver-protective effects.

### 6.9 Hypoglycemic effect

Natural products represent valuable sources for antidiabetic agents owing to their multi-target effects and safety (Khatoon et al., 2025). *I. cylindrica* polysaccharides have been demonstrated to improve glucose and lipid metabolism disorders in diabetic models, significantly reducing levels of glycated serum protein (GSP), triglycerides (TG), total cholesterol (TC), and low-density lipoprotein cholesterol (LDL-C), while increasing hepatic glycogen and high-density lipoprotein cholesterol (HDL-C) levels. The efficacy is comparable to that of metformin hydrochloride (Cui et al. 2012).

Hexokinase (HK) and pyruvate kinase (PK) are key rate-limiting enzymes in glycolysis. Their activities are significantly reduced under insulin resistance, making them important indicators for evaluating hypoglycemic effects *in vitro*. Further cell-based studies have demonstrated that the polysaccharide RPS-DS0.1 from *Imperata cylindrica* effectively enhances glucose consumption and glycogen synthesis in insulin-resistant HepG2 cells, elevates HK and PK activities, promotes glucose metabolism, and thus exerts hypoglycemic effects (Wei et al., 2024). In summary, *I. cylindrica* polysaccharides effectively improve glycolipid metabolism and insulin resistance by enhancing glucose consumption, glycogen synthesis, and the activities of HK and PK.

## 7 Toxicology

*I. cylindrica* has a long history as a medicinal and edible botanical drug. Acute oral toxicity studies in rats showed no mortality or adverse effects at 5,000 mg/kg for both aqueous and methanol rhizome extracts, confirming high acute safety (Chunlaratthanaphorn et al., 2007; Nayim et al., 2020). Subchronic studies found the water extract safe at up to 1,200 mg/kg/day for 90 days, whereas methanol extracts at 1,000 mg/kg/day caused mild hepatic degeneration, warranting caution with long-term non-aqueous extracts (Chunlaratthanaphorn et al., 2007; Nayim et al., 2020). The Chinese Pharmacopoeia (2025 Edition) classifies Imperatae Rhizoma as non-toxic, recommending 9–30 g/day of dried material.

Notably, while the rhizome is well-tolerated, *I. cylindrica* pollen is a clinically significant aeroallergen (Bijli et al., 2002)**.** It has been demonstrated that pollen processing critically affects allergenic potency: freeze-dried pollen stored at −70 °C retained 30 IgE-binding bands versus only 14 in oven-dried pollen, with ∼10-fold higher potency in ELISA inhibition assays. As the flower is also used medicinally (e.g., for hemostasis), pollen-related allergenicity warrants consideration in safety evaluations of whole-plant preparations, particularly for atopic individuals.

## 8 Ecology damage

*Imperata cylindrica* is a perennial grass that thrives in tropical and subtropical regions, originally ranging from Africa to southern Asia. The species has been extensively introduced beyond its native range and is now recognized as one of the world’s top ten most detrimental invasive plants (Redowan et al., 2025). A comprehensive dataset containing 6,414 occurrence records was employed in this study to simulate the species' ecological niche based on key climatic parameters such as temperature and soil moisture. The results indicated that over 16% of the global terrestrial surface is currently highly suitable for the growth of *I. cylindrica* (with an eco-climatic index ≥30), and Central America, Africa, and Australia have been identified as primary risk areas. Future projections based on the A2 scenario suggested that by 2050, 2080, and 2,100, the suitable habitats of this species will expand, especially in southern Argentina and parts of North America. The invasive potential of *I. cylindrica* is significantly influenced by climatic factors, particularly temperature and precipitation regimes. With the projected increase in global temperatures, this species is expected to expand its range into new regions, posing further challenges for ecosystem and natural resource management (Rad et al., 2025). It is regarded as a major weed of tea, rubber, pineapple, coconut., oil palm., and other perennial cash crops. It is also a dominant weed in the cultivation of cotton, soybean, maize, sugarcane, peanut, upland rice, and several vegetable crops (Haidar et al., 2021).

## 9 Comprehensive utilization

Its rapid spread and the consequent damage to agricultural production necessitate intensified relevant research, and the development of applications related to *I. cylindrica* is undoubtedly an effective measure. If this species can be extensively utilized in various industries including food, agriculture, animal husbandry, and ecological management, the large-scale demand generated therefrom will naturally curb its invasive tendency.

### 9.1 Applications in food

I. *cylindrica* exerts therapeutic effects on inflammatory diseases of the digestive, respiratory, and urinary systems. As recorded in *Compendium of Materia Medica*, this botanical drug is sweet in flavor, cold in nature, and non-toxic. It can treat heat stranguria characterized by painful and frequent urination, as well as dyspnea due to lung-heat. Studies have reported that the maximum tolerated dose (MTD) of high-concentration *I. cylindrica* and wax gourd tea in mice is 458 times the recommended daily dosage for humans (Luo et al., 2012). For toothpaste formulated with *I. cylindrica* extract, the median lethal dose (LD_50_) in mice is greater than 5 g/kg, which is actually classified as a non-toxic grade (Wei and Wang, 2024). Endowed with favorable safety profiles, it also exhibits satisfactory curative effects on minor ailments such as edema with oliguria and febrile diseases accompanied by polydipsia. Therefore, it serves as an important medicinal and edible botanical drug, boasting promising market prospects in the fields of health beverages and health foods, and can be processed into herbal teas, jellies, and various other food products.

#### 9.1.1 Beverages

A variety of beverages have been developed, showcasing its significant potential. These include: Imperatae Rhizoma and Honeysuckle Anti-fatigue Herbal Tea: Refreshing with a unique flavor, it also provides anti-fatigue effects (Wang et al., 2020); Imperatae Rhizoma and Chrysanthemi Flos (the flowers of *Chrysanthemum* × *morifolium* (Ramat.) Hemsl. [Asteraceae]) Beverage: This drink has a mild and pure taste, effectively retaining the original flavor and nutrients (Tang et al., 2018); Imperatae Rhizoma and Red Date (the fruit of *Ziziphus jujuba* Mill. [Rhamnaceae]) Compound Beverage: The aroma of dates blends with the natural fragrance of *I. cylindrica*, creating a distinct and layered flavor profile (Li et al., 2011). Other products include Imperatae Rhizoma and the leaves of *Lophatherum gracile* Brongn. [Poaceae] compound sports drinks (Ge, 2019), Imperatae Rhizoma and sugarcane beverages (Huang et al., 2006), and more.

#### 9.1.2 Jelly

Jellies using the rhizome of *I. cylindrica* as the main raw material, combined with compound gelling agents and erythritol in specific ratios, result in a product with a glossy appearance and a clear, sweet taste. The characteristic flavors of both components are prominent yet well-balanced, offering a complex taste experience with high overall acceptability (Chen et al., 2024). This provides a new option for consumers, particularly for specific groups with calorie control needs.

#### 9.1.3 Medicinal diet

*Imperata cylindrica* is one of the high frequency raw materials in the *Chinese medicinal diet dictionary*. It is often combined with other food and medicine homology ingredients to exert health-preserving or conditioning effects, ranking among the top 3 commonly used raw materials in boiling-type medicinal drinks and serving as one of the core ingredients in heat-clearing combinations (Wang and Xiang, 2024). Imperatae Rhizoma is often combined with ingredients such as Lonicerae Japonicae Flos (the flowers of *Lonicera japonica* Thunb. [Caprifoliaceae]), Phragmitis Rhizoma (the rhizome of *Phragmites australis* subsp. Australis [Poaceae]), and Mori Folium (the leaves of *Morus alba* L. [Moraceae]) to create health-preserving drinks with strong targeting. For example, boiling it together with Lonicerae Japonicae Flos and Chrysanthemi Flos can clear heat and reduce fire, soothe sore throat, and is suitable for drinking during summer heat or the initial stage of wind-heat invasion; when combined with Phragmitis Rhizoma and lotus root slices, it can enhance the effect of clearing heat and promoting fluid production, relieving discomfort such as dry mouth and tongue, restlessness and insomnia; decocting it with Artemisiae Scopariae Herba (the whole plant of *Artemisia scoparia* Waldst. and Kit. [Asteraceae] and *Artemisia capillaris* Thunb. [Asteraceae]) and Plantaginis Herba (the whole plant of *Plantago asiatica* L. [Plantaginaceae] and *Plantago depressa* Willd. [Plantaginaceae]) can strengthen the functions of inducing diuresis and eliminating dampness, making it particularly suitable for the daily conditioning of people with damp-heat constitution.

#### 9.1.4 Others

Additionally, patents have been filed for other products such as Imperatae Rhizoma preserved fruit (CN105558235A), Imperatae Rhizoma noodles (CN1711896) and Imperatae Rhizoma health vermicelli (CN1826958). These innovations demonstrate its potential value in a wider range of food applications, awaiting further exploration by researchers.

### 9.2 Agricultural and animal husbandry value

*Imperata cylindrica* has various animal husbandry value. Research shows that it has a growth-promoting effect on meat rabbits. Adding fresh rhizome to feed reduced the diarrhea rate of meat rabbits, thereby improving their production performance (Zhang, 2012). At the same time, the oligosaccharides contained in its rhizome effectively balance the intestinal microflora, which is conducive to the digestion and absorption of nutrients and improved the milk production performance of dairy cows (Wang and Zhao, 2012). Therefore, *I. cylindrica* has certain application prospects in animal husbandry, contributing to economic development. A treatment with its rhizome cold water extract significantly reduced the spoilage rate of strawberries during storage. It effectively delayed the loss of nutrients and the decline in quality, performing comparably to conventional preservatives (Jiang, Zhu, and Li, 2011). This finding represents an important advancement for preserving strawberries, a fruit with highly demanding freshness requirements. Extracts from its rhizome can effectively reduce the damage caused by Phytophthora infestans to potatoes, with efficacy comparable to that of products from biological agent manufacturers (Dyah et al., 2024). The stems and leaves of *I. cylindrica* contain 42.33% cellulose, with an average fiber length of 28.57 mm. The fiber yield from stems and leaves is 6.67%. Beyond their use in papermaking and weaving, natural polymers such as cellulose, hemicellulose, and lignin can be extracted from them for the production of pharmaceutical and chemical products like xylitol and fuel ethanol (Deng, 2009). As a widely distributed plant, the full and effective utilization of its stems and leaves would undoubtedly generate significant economic value.

### 9.3 Ecological value

*Imperata cylindrica* exhibits allelopathic effects on surrounding plants, attributable to metabolites such as 5-methoxyflavone and 5,20-dimethoxyflavone. This allelopathic potential positions it for future development in the field of herbicides (Suzuki et al., 2018). Regarding copper ions in contaminated soil, *I. cylindrica* can tolerate high copper exposure by increasing the synthesis of phenolic metabolites in its shoots and accumulating a substantial amount of copper in its rhizome. This indicates its potential value for phytoremediation applications in copper-polluted environments (Vidal et al., 2020). *I. cylindrica* also demonstrates a certain absorption capacity for lead in soil, showing potential as a soil remediation agent (Paz-Alberto et al., 2007).This plant could be used to remediate metal tailings contaminated by heavy metals, especially for areas with high Mn Cd and Ni pollution (Song et al., 2019).In terms of water purification, two types of nickel ion adsorbents prepared using Imperata Rhizome as a raw material have demonstrated effective adsorption of nickel ions in water bodies (Li et al., 2013).The collective evidence positions *I. cylindrica* as a highly effective plant-based remediator for heavy metals in polluted ecosystems. Additionally, original liquid extract of Imperata Rhizome can promote the rooting of pine cuttings (Cao, 1987). In summary, *I. cylindrica* demonstrates significant ecological value across multiple diverse application fields.

## 10 Summary and outlook

*I. cylindrica* is both an invasive species and a valuable botanical resource, making it an important subject for interdisciplinary research. Although it is considered an invasive species that threatens agricultural production and ecosystem stability, its abundant chemical metabolites, diverse pharmacological activities, and broad application potential in medicine, food, agriculture, and ecological restoration have been widely demonstrated.

However, current research remains predominantly descriptive rather than mechanistically analytical. Although 141 chemical metabolites have been identified, most pharmacological studies are based on crude extracts rather than purified active monomers. High-quality clinical trials are lacking, and most evidence is derived from *in vitro* assays or small-animal models with limited translational relevance. Furthermore, research is heavily concentrated on the rhizome, whereas aerial parts remain under-explored. Methodological inconsistencies, including insufficient standardization of herbal materials, small sample sizes, inadequate controls, and low reproducibility, further restrict the reliability and comparability of existing findings.

Based on the comprehensive review, we conclude that *I. cylindrica* possesses credible traditional medicinal value, definite phytochemical diversity, and promising biological activities, yet its modern development is hindered by insufficient mechanistic research, inadequate clinical evidence, and incomplete resource utilization.

To promote its rational development and ecological management, future research directions are proposed as follows:

The highest priority for follow-up research is to establish high-level clinical evidence; large-sample, multi-center randomized controlled trials targeting the traditional indications of *Imperata cylindrica* including inflammatory disorders, kidney diseases and hemorrhagic conditions should be launched, with unified observation indicators, medication regimens and long-term follow-up standards formulated to verify its *in-vivo* efficacy, applicable dosage range and long-term safety in human subjects, which lays an indispensable foundation for its clinical promotion and transformation. On this basis, systematic activity screening of all reported chemical constituents of *I*. *cylindrica* needs to be implemented to pinpoint key bioactive monomers corresponding to diverse pharmacological functions, alongside comparative efficacy analysis among different extracts, fractions and single metabolites and exploration of synergistic or antagonistic interactions between multiple ingredients so as to fully clarify its definitive pharmacodynamic material basis.

Next, experimental systems should be optimized comprehensively: clinically relevant chronic disease models such as chronic inflammation and progressive renal fibrosis ought to be adopted to replace overused acute animal models that fail to recapitulate long-term pathological progression of human illnesses, and multi-gradient dose groups should be arranged uniformly in subsequent *in vivo* and *in vitro* experiments to generate complete dose-response curves and define minimum effective doses and safe dose windows, greatly improving the reproducibility and rigor of experimental design. Afterwards, in-depth systematic mechanistic research is required to dissect the upstream and downstream regulatory networks of core signaling pathways involved in anti-inflammatory and renoprotective effects, interpret the crosstalk between different pathways, inflammatory response, oxidative stress and intestinal flora metabolism, and conduct structure-activity relationship analysis of characteristic active monomers to supply theoretical support for subsequent structural modification and new drug development.

Finally, full-spectrum safety evaluation and methodological standardization need to be completed step by step, including comprehensive acute, subchronic and chronic toxicity tests and drug interaction assessments to build a complete safety evaluation framework; rigorous blank controls and counter-screening assays must be incorporated to eliminate non-specific interference and false-positive outcomes triggered by complex phytochemical compositions, while unified extraction procedures, detection indicators and evaluation criteria will be formulated to standardize the whole research system of this medicinal plant.

Building upon the standardized pharmacological research framework outlined above, further supporting technical optimization and industrial translation work can be carried out stepwise. A unified, standardized quality evaluation system for *I. cylindrica* raw materials can be formulated, covering geographical origin tracing, harvesting timing, processing procedures and chemical fingerprint profiling, so as to guarantee consistent quality across different production batches. In addition, phytochemical and pharmacological research, which has long focused merely on rhizomes, ought to be extended to aerial tissues including leaves, stems and flowers, enabling full-plant resource exploitation and the creation of value-added derivatives. Considering the invasive traits of this species, ecological modeling and molecular ecological analyses coupled with invasiveness mechanism exploration can be integrated to build an invasion risk assessment and early warning platform, which balances sustainable resource exploitation and biological invasion management. Relying on the validated pharmacodynamic evidence obtained from basic research, multiple high-value-added products, such as novel pharmaceutical preparations, functional foods, natural food preservatives and phytoremediation reagents, can be developed to achieve full industrial conversion of *I. cylindrica* resources. Overall, future research should move beyond simple descriptive literature collation and shift toward mechanism-oriented, standardized and clinically verified research systems. These multi-layered research strategies will help convert *I. cylindrica* from an underexploited invasive weed into a well-supported medicinal and industrial resource with solid scientific backing.
